# Putting the Pieces Together: Mental Construction of Semantically Congruent and Incongruent Scenes in Dementia

**DOI:** 10.3390/brainsci12010020

**Published:** 2021-12-24

**Authors:** Nikki-Anne Wilson, Rebekah M. Ahmed, Olivier Piguet, Muireann Irish

**Affiliations:** 1 Brain and Mind Centre, The University of Sydney, Sydney, NSW 2050, Australia; n.wilson@neura.edu.au (N.-A.W.); rebekah.ahmed@sydney.edu.au (R.M.A.); olivier.piguet@sydney.edu.au (O.P.); 2 School of Psychology, The University of Sydney, Sydney, NSW 2006, Australia; 3 School of Psychology, The University of New South Wales, Sydney, NSW 2052, Australia; 4 Neuroscience Research Australia, Sydney, NSW 2031, Australia; 5 Memory and Cognition Clinic, Institute of Clinical Neurosciences, Royal Prince Alfred Hospital, Sydney, NSW 2050, Australia

**Keywords:** scene construction, schema, semantic memory, episodic memory, frontotemporal dementia, imagination, social cognition

## Abstract

Scene construction refers to the process by which humans generate richly detailed and spatially cohesive scenes in the mind’s eye. The cognitive processes that underwrite this capacity remain unclear, particularly when the envisaged scene calls for the integration of various types of contextual information. Here, we explored social and non-social forms of scene construction in Alzheimer’s disease (AD; *n* = 11) and the behavioural variant of frontotemporal dementia (bvFTD; *n* = 15) relative to healthy older control participants (*n* = 16) using a novel adaptation of the scene construction task. Participants mentally constructed detailed scenes in response to scene–object cues that varied in terms of their sociality (social; non-social) and congruence (congruent; incongruent). A significant group × sociality × congruence interaction was found whereby performance on the incongruent social scene condition was significantly disrupted in both patient groups relative to controls. Moreover, bvFTD patients produced significantly less contextual detail in social relative to non-social incongruent scenes. Construction of social and non-social incongruent scenes in the patient groups combined was significantly associated with independent measures of semantic processing and visuospatial memory. Our findings demonstrate the influence of schema-incongruency on scene construction performance and reinforce the importance of episodic–semantic interactions during novel event construction.

## 1. Introduction

Scene construction refers to the capacity to generate and maintain a richly detailed, spatially cohesive mental scene, and relies on coordinated activity within a distributed brain network centred on the hippocampus [1,2,3]. Previous reports have identified associations between scene construction and autobiographical memory [4], future thinking [5], and navigation [6]. Theory of Mind (ToM), or mentalising, has also been theorised to depend on the content and phenomenological quality of mentally constructed scenes [7]. Collectively, these findings have garnered support for the view that scene construction forms the foundation for an array of higher-order complex constructive endeavours [8]. While significant advances have been made in delineating the neural bases of scene construction, comparatively less is known regarding the cognitive processes that underwrite this capacity, particularly when the envisaged scene calls for the integration of various types of contextual information.

Episodic memory and scene construction are closely connected, as reflected by the significant overlap of their neural substrates [1,5] and parallel impairments observed in clinical populations ([9], but see [10]). By contrast, less is known regarding the potential role of semantic memory in the construction of mental scenes. Initial evidence from developmental amnesic patients hinted at the possibility that a residual capacity for scene construction is supported by intact semantic memory [11,12]. These findings have led to greater consideration of the role of conceptual knowledge in the constructive endeavour [8,13,14]. Empirical demonstrations of the pivotal role of semantic memory in past and future forms of mental construction [15] have further fuelled the debate regarding the interplay between episodic and semantic elements during mental construction [16,17,18]. Accordingly, semantic memory is proposed to provide the requisite scaffold or organisational framework to guide retrieval of past events, the simulation of future scenarios, and the mental representation of spatially coherent scenes [16,19]. With an appropriate semantic framework in place, details from episodic experiences, along with conceptual information, and event scripts can then be assimilated into the simulation to create a detailed and spatially integrated mental representation [14,18].

Another way by which semantic memory may support the constructive endeavour is in supporting knowledge manipulation and generalisation during the construction of new experiences [20]. Indeed, this form of conceptual association is well-documented in the creativity literature, enabling access to relevant conceptual information and the ability to draw appropriate links between concepts to generate novel ideas [21]. In this regard, semantic elements or objects—their number, form, and their inter-relationships—appear to heavily moderate how a mental scene is constructed. For example, envisaging three objects within a three-dimensional space is sufficient to evoke the subjective experience of a real-world scene [22]. In contrast, mentally generating three objects within a two-dimensional space fails to evoke the same subjective experience of a scene [23]. The nature of an object also contributes to how a scene is constructed, with space-defining objects (e.g., a wooden rocking chair) more central and evoking a greater sense of spatial context than space-ambiguous objects (e.g., a folded newspaper; [24]). As such, the relationship between scenes and objects, and the conceptual categories they invoke, plays a significant role in the way in which a mental scene is constructed and subjectively experienced.

Objects are, of course, not the only items that define a scene. The social nature of scenes, for example, requires the curation, selection, and integration of social elements, i.e., people, into the spatial array [25,26]. If different objects evoke different levels of spatial context, the inclusion of people, as a social class of object, likely requires the added consideration of the thoughts and emotions of an envisaged person, as well as their actions and interactions within the scene [25]. Envisaging social scenes has been shown to more heavily draw upon previous experience than constructing non-social scenes [26], resonating with suggestions of a foundational role for episodic memory in prosocial behaviour [27].

Finally, it is important to consider whether scene and object information is congruent with pre-existing knowledge structures. Schemas are superordinate knowledge structures that extract commonalities across events and experiences [28] and are suggested to provide the necessary framework from which a scene is created [29,30]. Once activated, schema templates influence how incoming information is processed whereby congruent information is prioritised and strengthened, while incongruent information may be deemed inconsequential and discarded [28]. Empirical studies in healthy adults indicate that information that is congruent with existing schemas is recognised and recollected more accurately and more quickly relative to incongruent information (reviewed by [31]). How schema incongruency influences the construction and quality of mental scenes remains unclear.

Given these intersecting lines of enquiry, the current study sought to explore how object–scene (in)congruency impacts the capacity for scene construction in dementia syndromes characterised by variable impairments in scene construction, semantic memory, and social cognition. The behavioural variant of frontotemporal dementia (bvFTD) is a younger-onset neurodegenerative disorder characterised by profound changes in personality and behaviour, executive dysfunction, and disinhibition, resulting in severe socioemotional dysfunction [32,33]. We have previously demonstrated that patients with bvFTD exhibit profound impairments in the construction of future scenarios [34] and commonplace scenes [35], which is exacerbated when the scene calls for the integration of social information [26]. In contrast, patients with Alzheimer’s disease (AD) typically present with episodic memory disturbances alongside visuospatial dysfunction and, to a lesser extent, semantic processing difficulties [36]. Recent studies indicate marked impairments in AD across an array of constructive processes including future thinking [37,38] and scene construction [39] in the context of relatively preserved social cognition [40,41]. Given these differential profiles of loss and sparing, these syndromes provide a unique opportunity to explore the integration of semantically congruent and incongruent objects within scene arrays that vary in terms of their social nature. As such, we manipulated the semantic congruency of object–scene pairings along with the sociality of the to-be-constructed scene with the hypothesis that incongruency would disrupt the constructive endeavour most prominently for social scenes in bvFTD.

## 2. Materials and Methods

### 2.1. Participants

Fifteen individuals with a clinical diagnosis of probable bvFTD and 11 individuals with typical Alzheimer’s disease (AD) were contrasted with 16 healthy older control participants. Briefly, clinical presentation of bvFTD included progressive behavioural and/or personality changes including inappropriate behaviour, apathy, reduced empathy, perseverative behaviour and/or executive dysfunction [32]. Conversely, AD patients presented with significant episodic memory, visuospatial, and language (particularly word-finding) difficulties, in the context of relatively intact social behaviour [36]. Participants were recruited through FRONTIER, the frontotemporal dementia research group based at the Brain and Mind Centre, The University of Sydney. Dementia diagnosis was based on multidisciplinary consensus incorporating clinical history, cognitive profile, and neuroimaging. Previous neurological or neuropsychiatric conditions, substance abuse or a lack of proficiency in English precluded participation in the study. Dementia patients were excluded if they achieved a score lower than 40 on the Addenbrooke’s Cognitive Examination III (ACE-III) due to the severity of their cognitive impairment. Control participants were required to score >88 on the ACE-III. The Frontotemporal Lobar Degeneration-modified Clinical Dementia Rating scale (CDR-FTLD) Sum of Boxes score [42] was used as an index of disease severity, while the Cambridge Behavioural Inventory-Revised (CBI-R) [43] provided a measure of behavioural changes, as rated by the informant.

### 2.2. Cognitive Assessment

All participants completed a comprehensive neuropsychological battery assessing the main cognitive domains as well as global cognitive function (ACE-III; [44,45]). Verbal episodic memory was assessed using the Rey Auditory Verbal Learning Test (RAVLT; [46]), while nonverbal episodic memory was measured using the 3 min delayed recall of the Rey Complex Figure (RCF; [47]). Language abilities were evaluated using targeted assessments of naming, comprehension, and semantic association from the Sydney Language Battery (SydBAT; [48]). Measures of executive function included Digit Span Forwards and Backwards [49] to index attention and working memory, respectively, as well as the time difference between parts B and A on the Trail Making Test (TMT; [50]) and the scaled score on the Hayling Sentence Completion Test [51].

### 2.3. Scene Construction Task

A modified version of the scene construction task [52] was used with sociality and congruence manipulated. Briefly, participants imagined and described aloud atemporal scenes in as much detail as possible, avoiding restating a memory. Each scene cue contained a background setting and a person or object, dependent on the level of sociality (i.e., people in the social conditions, objects in the non-social conditions). The background setting was either congruent or incongruent with the person or object. Congruent trials included hospital (background scene) and doctor (person, social); and classroom (background scene) and books (object, non-social). Congruency was informed by ratings of which items were most likely to go together from pilot testing in a sample of healthy young adults (*N* = 10; see Supplementary Material). Incongruent trials represented combinations least likely to go together, including funeral (background scene) and clown (person, social); and beach (background scene) and ice-skates (object, non-social). A 2 × 2 design was used exploring Congruency (congruent, incongruent) and Sociality (social, non-social). To minimise risk of fatigue and cognitive demand in dementia patients, each scene description was limited to 2 min. Congruent and incongruent trials were completed in a blocked design, with order of blocks counterbalanced across participants.

The current study used succinct scene cues, which were standardised in syntactic structure (e.g., “You’re at a funeral. There is a clown there”) across conditions to limit cognitive demand and the possibility of unintentionally probing inter-item relationships. Participants were instructed to make sure to include both elements (i.e., object/person and background) into a coherent scene description, “Even if the two things don’t feel like they belong together at all, I want you to try really hard to create as believable a scenario as possible including both the background setting and the person or object.” An example of an incongruent scene was provided (Office Boardroom and Hairdryer) whereby the experimenter confirmed with the participant that the two example elements did not go together but then pretended to complete the task while reciting a standardised scene description. Cues were read aloud and presented on a sheet of paper, which remained in front of participants for the duration of the trial to minimise working memory demands. General prompts were provided to encourage elaboration or if the participant failed to include the two scene elements (see Supplementary Material for the example scene and prompts). Importantly, these prompts were non-directive, limited to two per scene and merely served to encourage the participant to provide additional details. For example, “Remembering to include both the X and the Y in the scenario that you’re describing, are there any other details you can tell me?” The entire test session lasted approximately 25–30 min and was digitally recorded for subsequent transcription and scoring.

### 2.4. Subjective Ratings

In keeping with the original Hassabis et al. protocol [52], following each scene description, participants were asked to rate the constructed scene in terms of perceived difficulty, vividness, level of detail, sense of presence, and similarity to a previous memory. An additional rating was included to capture the subjective degree to which the two elements were realistically integrated into a coherent scene, “How realistic did the scene feel to you?”, rated on a scale from 1 to 5, with higher ratings indicating a stronger sense of realism.

### 2.5. Scoring

Total Content scores represented the primary measure of scene construction performance. Scene transcripts were segmented into discrete contextual detail types: (i) Entities Present, (ii) Sensory Descriptions, (iii) Spatial References, and (iv) Thoughts/Emotions/Actions (see [52] for full scoring details). The maximum number of details for each subcategory was capped at 7 points, leading to a maximum Total Content score of 28, in keeping with the original scene construction scoring protocol [52].

### 2.6. Statistical Analyses

Data were analysed using IBM SPSS version 26. For continuous variables, normality of distributions was examined using Kolmogorov–Smirnov tests. Group differences for normally distributed continuous variables (e.g., age at assessment, years of education) were assessed using univariate ANOVAs. Group differences on categorical variables (e.g., sex) were examined using Chi-squared tests. Where limited cognitive data resulted in small and uneven sample sizes, or data were non-normally distributed (e.g., participant subjective ratings), non-parametric Kruskal–Wallis tests for independent samples and Wilcoxon signed-rank tests for related samples were used. Group differences on the scene construction task were assessed via a mixed 3 × 2 × 2 ANOVA with group (Control, AD, bvFTD) as the between-subjects factor, and congruency (congruent, incongruent) and sociality (social, non-social) as the within-subject factors. For ease of interpretation, and due to no main effect of congruency being found, two mixed 3 × 2 × 4 ANOVAs were performed in the incongruent and congruent conditions separately with group as the between-subjects factor, and sociality (social, non-social) and contextual detail category (Entities Present, Sensory Descriptions, Spatial References and Thoughts/Emotions/Actions) as the within-subject factors. Post hoc comparisons were adjusted using Bonferroni correction where appropriate; however, due to the exploratory nature of the study, where extensive comparisons would have resulted in overly conservative Bonferroni correction (e.g., subjective ratings, correlations with cognitive variables), uncorrected values are reported. The alpha level to determine statistical significance was set at *p* < 0.05. Partial eta-squared values ($\eta_{p}^{2}$) were assessed as a measure of effect size for ANOVA statistics.

## 3. Results

### 3.1. Demographic and Clinical Information

Age at assessment, F(2, 39) = 1.0; *p* = 0.393; η_p_^2^ = 0.05, and sex distribution, χ^2^(2, 42) = 5.15; *p* = 0.076, did not differ significantly across Control, AD and bvFTD groups (Table 1). Years of education, however, differed significantly across groups, F(2, 38) = 8.03; *p* = 0.001; η_p_^2^ = 0.30, driven by higher levels of education in Controls relative to the two dementia syndromes (both *p* values ≤ 0.038). Level of education was comparable between the two patient groups (*p* = 0.707). Controlling for years of education did not change the significant three-way interaction for Total Content scores and, thus, education was not considered further in the analyses. Disease severity (CDR-FTLD SoB) and duration (years from symptom onset), t(21) = 1.57, *p* = 0.131, were comparable between the two patient groups (both *p* values > 0.13). Finally, a significant group effect for overall behavioural change was found (CBI-R), F(2, 36) = 21.32; *p* ≤ 0.0001; η_p_^2^ = 0.54. Bonferroni-adjusted post hoc tests showed comparable overall carer-rated behavioural changes in the patient groups (*p* = 0.517); however, bvFTD patients were rated as exhibiting more abnormal behaviours relative to the AD group (*p* = 0.046).

### 3.2. Cognitive Profiles

Relative to Controls, bvFTD and AD patients displayed characteristic cognitive deficits largely in keeping with their clinical diagnoses (Table 2). Significant group effects emerged across all cognitive variables (all *p* values ≤ 0.007) with patients scoring significantly worse than Controls for response inhibition and working memory (Hayling, bvFTD, *p* = 0.013, AD, *p* ≤ 0.0001; Digit Span Backwards, bvFTD, *p* = 0.039, AD, *p* = 0.001), delayed verbal episodic memory (RAVLT 30 min, bvFTD, *p* = 0.001, AD, *p* < 0.0001), and verbal fluency (bvFTD, *p* = 0.001, AD, *p* ≤ 0.0001). Compared to Controls, both patient groups showed significant deficits in overall language ability (ACE Language, bvFTD, *p* = 0.006, AD, *p* ≤ 0.0001), with semantic association (bvFTD, *p* = 0.243, AD, *p* ≤ 0.0001) and naming (bvFTD, *p* = 0.145, AD, *p* ≤ 0.0001) impairments on the SydBat occurring exclusively in the AD group. AD patients further displayed marked visuospatial episodic memory dysfunction (RCF 3 min, *p* ≤ 0.0001), along with deficits in attention (Digit Span Forwards, *p* = 0.007) and divided attention (TMT B-A, *p* = 0.003) relative to Controls. These impairments were not evident in the bvFTD group (all *p* values ≥ 0.05). Direct comparison of the patient groups revealed disproportionate deficits in visuospatial episodic memory in the AD group relative to the bvFTD group (RCF 3 min: *p* = 0.005).

### 3.3. Scene Construction Performance

#### 3.3.1. Total Content

A significant group effect in terms of content was evident on the scene construction task, F(2, 39) = 53.04, *p* ≤ 0.0001, η*_p_*^2^ = 0.73. Post hoc tests revealed that total content scores were significantly lower for both AD and bvFTD groups compared to control participants (both *p* values ≤ 0.0001), with no significant difference between the patient groups (*p* = 0.481). A main effect of sociality, F(1, 39) = 18.58, *p* ≤ 0.0001, η*_p_*^2^ = 0.32, was also present, with participants performing more poorly overall on social, relative to non-social, scenes (*p* ≤ 0.0001). No main effect of congruency was found, F(1, 39) = 2.53, *p* = 0.120, η*_p_*^2^ = 0.06.

**Figure 1:**
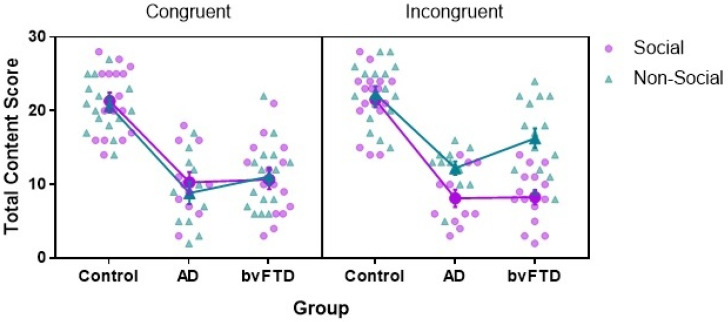
Group performance on social and non-social scene construction in congruent and incongruent conditions. Interaction based on estimated marginal means of average content score (max 28) with whiskers representing standard error of measurement. Data points show individual scores. AD = Alzheimer’s disease. bvFTD = behavioural variant of frontotemporal dementia.

A significant group × sociality × congruency interaction was observed, F(2, 39) = 3.52; *p* = 0.039, η*_p_*^2^ = 0.15 (Figure 1). This was qualified by a significant sociality × congruency interaction, F(1, 39) = 23.03; *p* ≤ 0.0001, η*_p_*^2^ = 0.37, whereby significantly more content was generated for non-social relative to social scenes in the incongruent condition (*p* ≤ 0.0001; congruent: *p* = 0.417), irrespective of group membership. Closer inspection of the incongruent condition revealed that both patient groups performed significantly better on non-social, relative to social, trials (AD *p* = 0.002; bvFTD *p* ≤ 0.0001). This effect was not observed in the congruent condition (all *p* values ≥ 0.2). A significant group × sociality interaction, F(2, 39) = 9.00; *p* = 0.001; η*_p_*^2^ = 0.32, was also found. Post hoc tests showed that bvFTD patients generated significantly more detailed non-social, relative to social, scenes (*p* ≤ 0.0001), irrespective of congruency. No such effect was observed for AD patients or Controls (both *p* values ≥ 0.1). Irrespective of sociality, both patient groups performed significantly worse than Controls at each level of congruency (all *p* values ≤ 0.0001), with no significant difference between the patient groups (both *p* values > 0.39). Finally, no significant group × congruency interaction was found, F(2, 39) = 0.14; *p* = 0.870; η*_p_*^2^ = 0.01.

#### 3.3.2. Contextual Detail Profile

Two mixed 2 × 4 × 3 ANOVAs were performed in the incongruent and congruent conditions separately to examine group differences in contextual details generated across social and non-social conditions. Main effects, interactions and post hoc comparisons are reported for incongruent and congruent analyses separately.

In the incongruent condition, a significant group effect was found, *F*(2, 39) = 42.68; *p* ≤ 0.0001, η*_p_*^2^ = 0.69, with Controls outperforming the AD and bvFTD patient groups (both *p* values ≤ 0.0001) and no difference between the patient groups (*p* = 0.358). A significant main effect of sociality was evident, *F*(1, 39) = 46.46; *p* ≤ 0.0001; η*_p_*^2^ = 0.54, whereby scene descriptions were significantly more detailed in non-social, relative to social, scenes (*p* ≤ 0.0001), irrespective of detail type or group membership. Finally, a significant main effect of detail type was found, *F*(3, 37) = 4.54; *p* = 0.008; η*_p_*^2^ = 0.27. Relative to the other detail categories, participants produced significantly more unique entities relative to sensory details (*p* = 0.022) and spatial references (*p* = 0.016).

**Figure 2:**
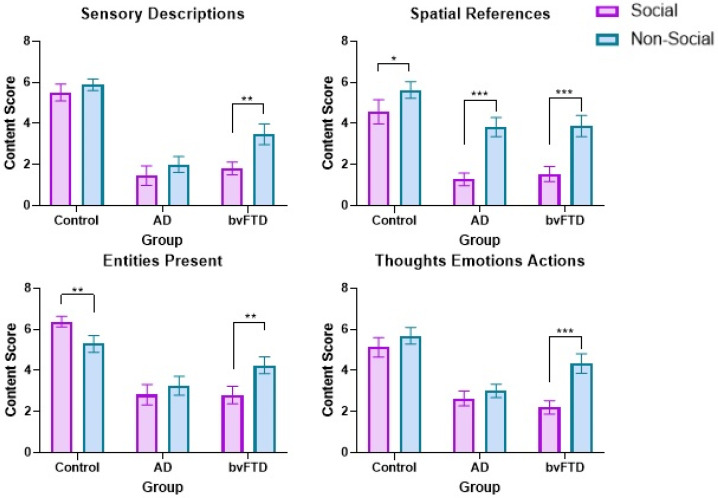
Mean social and non-social scene construction contextual detail scores in the incongruent condition. Whiskers represent standard error of measurement. AD = Alzheimer’s disease. bvFTD = behavioural variant of frontotemporal dementia. * = *p* < 0.05, ** = *p* < 0.01, *** = *p* ≤ 0.0001.

A significant sociality × group interaction, *F*(2, 39) = 12.67; *p* ≤ 0.0001, η*_p_*^2^ = 0.39, showed that, irrespective of detail type, both patient groups performed significantly better in the non-social, relative to social, condition (AD, *p* = 0.002; bvFTD, *p* ≤ 0.0001; Figure 2), with no such difference observed in the Control group (*p* = 0.419). Finally, a significant sociality × detail interaction, *F*(3, 37) = 6.75; *p* = 0.001; η*_p_*^2^ = 0.35, reflected the fact that, irrespective of group membership, participants provided more sensory descriptions, spatial references and thoughts, emotions and actions (but not entities present, *p* = 0.271) in the non-social, relative to social, conditions (all *p* values ≤ 0.004). The three-way group × sociality × detail interaction for incongruent scenes was not significant, *F*(6, 76) = 1.58; *p* = 0.166; η*_p_*^2^ = 0.11.

Considering next the congruent condition, a significant group effect was found, *F*(2, 39) = 36.19; *p* ≤ 0.0001; η*_p_*^2^ = 0.65, with both AD and bvFTD patient groups performing significantly worse than Controls (both *p* values ≤ 0.0001), while performance between patient groups was comparable (*p* = 0.799). No significant main effect for sociality emerged*, F*(1, 39) = 0.67; *p* = 0.417; η*_p_*^2^ = 0.02; however, a significant main effect of detail was found, *F*(3, 37) = 9.92; *p* ≤ 0.0001; η*_p_*^2^ = 0.45. Post hoc comparisons showed that, irrespective of group membership, participants produced significantly more sensory details relative to spatial references (*p* = 0.024) and thoughts, emotions, and actions (*p* = 0.007), and more unique entities relative to spatial references (*p* = 0.008).

**Figure 3:**
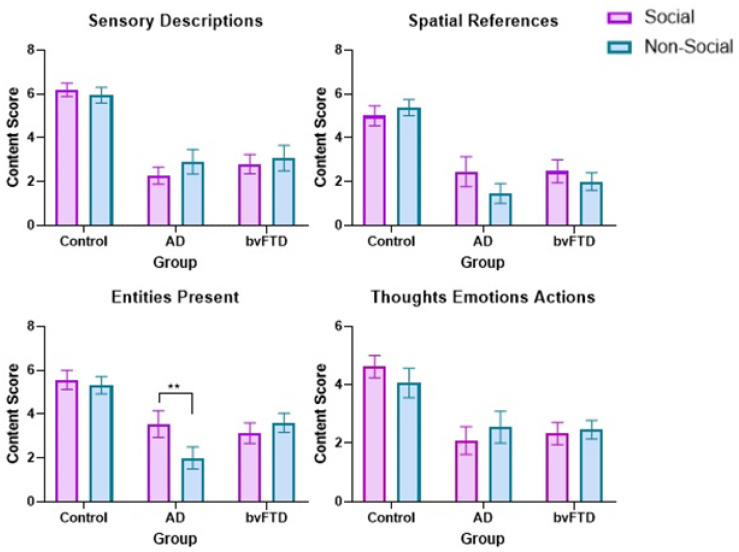
Mean social and non-social scene construction contextual detail scores in the congruent condition. Whiskers represent standard error of measurement. AD = Alzheimer’s disease. bvFTD = behavioural variant of frontotemporal dementia. ** = *p* < 0.01.

Importantly, a significant three-way sociality × detail × group interaction was found in the congruent condition, F(6, 76) = 2.63; *p* = 0.023; η*_p_*^2^ = 0.17. Post hoc tests showed that AD patients provided significantly fewer entities present in the non-social, relative to the social, condition (*p* = 0.009), with a similar trend observed for spatial references (*p* = 0.055, Figure 3). No other significant differences in content scores were present between social and non-social conditions in any of the groups (all *p* values ≥ 0.2). None of the two-way interactions were significant: sociality × group, *F*(2, 39) = 0.55; *p* = 0.579; η*_p_*^2^ = 0.03, sociality × detail, *F*(3, 37) = 1.09; *p* = 0.364; η*_p_*^2^ = 0.08, detail × group, *F*(6, 76) = 1.39; *p* = 0.230; η*_p_*^2^ = 0.10.

#### 3.3.3. Participant Subjective Ratings

We next explored whether participant subjective ratings differed according to the social nature of the constructed scene and the congruency of scene–object cues. Independent samples Kruskal–Wallis tests failed to reveal any significant group differences in terms of subjective ratings of overall difficulty, vividness, level of detail, sense of presence, perceived realism, or similarity to a previous memory (all *p* values ≥ 0.19).

Two sets of Wilcoxon signed-rank tests were used to explore phenomenological differences in the construction of social and non-social scene types within each group separately (Table 3). BvFTD (*Z* = −2.52, *p* = 0.012) and AD (*Z* = −2.23, *p* = 0.026) patients rated the construction of social scenes based on congruent scene–object pairs as more similar to a previous memory than scenes based on incongruent pairings. The bvFTD group further rated the construction of social scenes based on incongruent scene–object pairs as more difficult compared to congruent pairs (Z = −2.23, *p* = 0.026). All participant groups rated the construction of non-social scenes based on congruent scene–object pairs as more similar to a previous memory than those based on incongruent scene–object pairs (bvFTD: Z = −2.70, *p* = 0.007; AD: Z = −2.23, *p* = 0.026; Control: Z = −2.72, *p* = 0.006). No other comparisons were significant (all *p* values ≥ 0.07).

### 3.4. Correlations between Scene Construction and Selected Cognitive Variables

One-tailed Pearson correlations were run to explore potential associations between Total Content generated for each condition and performance on selected measures of cognitive function in the patient groups combined (*n* = 23; see Table 4). Measures of semantic processing on the SydBat were moderately associated with congruent and incongruent non-social scene construction (all r values ≥ 0.3), while response inhibition was associated with congruent social scene construction. Delayed visuospatial episodic recall (RCF 3 min recall) was found to correlate with non-social scene construction performance in both the congruent and incongruent conditions (all r values ≥ 0.4).

## 4. Discussion

The objective of this study was to explore how schema congruency influences the generation of contextual details and the accompanying subjective experience during scene construction in dementia. Using a novel extension of the classic scene construction paradigm, we manipulated the congruency of scene–object cues across social and non-social contexts. Overall, we observed differential effects of congruency on the capacity for social versus non-social scene construction, with incongruent social scenes disproportionately affected in dementia. This effect was not observed in the congruent condition, with comparable performance across social and non-social trials within each group. We consider the potential underlying mechanisms that drive disproportionate impairments in the construction of incongruent social scenes as well as possible clinical implications of such impairments for people with dementia.

The most striking finding in this study is our observation of profound impairments in the construction of social scenes that require the integration of incongruent scene–object cues. Previous studies have demonstrated a grossly diminished capacity for mental construction in bvFTD spanning episodic and autobiographical memory [53,54,55,56], episodic and semantic forms of future simulation [34,57], and the construction of commonplace atemporal scenes [35]. Importantly, we replicated our previous finding of markedly compromised social relative to non-social scene construction in bvFTD [26], but extended these findings by considering the modulating role of scene–object congruency on the constructive endeavour. Within-group analyses revealed that bvFTD patients generated significantly fewer contextual details on social relative to non-social trials, most pronounced for the incongruent condition. This finding indicates a specific impairment in the construction of social scenes, which call for the integration of elements that are incompatible with existing schemas. Notably, bvFTD patients subjectively rated incongruent social scenes as more difficult to construct relative to congruent social scenes, suggesting a convergence between objective task performance and phenomenology in this group.

We tentatively interpret these findings as reflecting the higher integrative load of the incongruent social condition, whereby participants are required to combine two semantically unrelated items (e.g., clown, funeral) that are highly unlikely to co-occur within the same social context. Previous work suggests that increasing the constructive demands of future simulation tasks is associated with significantly reduced episodic detail in older adults [58]. Using a novel experimental task, Addis and colleagues manipulated the recombinatorial load of stimulus sets comprising person, place, and object details for past and future conditions. Events requiring the integration of person, place, and object details taken from three separate events were found to be less detailed and less rich in terms of phenomenology in older adults. Moreover, events simulated under high recombinatorial load were rated as less similar to previous memories, suggesting the events generated were highly novel and not likely to have been previously experienced [58]. Looking at the similarity to past memory ratings provided by participants in the current study, we found that congruent social scenes were rated by bvFTD and AD patients as more similar to a previous memory than incongruent social scenes. It may be that congruent social scenes (in this case “doctor– hospital”) more readily evoke well-defined event scripts (e.g., what usually happens in the hospital) or personally experienced memories (“the last time I was in hospital”) that support the construction of a unified spatial array [59,60,61]. In contrast, incongruent scenes (“clown–funeral”) require the integration of details that do not typically coincide within a given spatial or social context, thus precluding the ability to draw upon previous experiences. Such novel events have been shown to rely more heavily upon semantic, rather than episodic, memory [62], suggesting a compensatory mechanism when episodic content is low [63].

Our correlation analyses revealed significant associations between the construction of congruent and incongruent non-social scenes in the combined patient group and independent measures of semantic processing and delayed visuospatial episodic recall. The semantic association task measures the capacity to bridge disparate semantic concepts via an appropriate semantic link, providing an index of semantic relational processing, while the visuospatial task assesses delayed episodic memory retrieval for non-verbal material. Our finding of comparable associations between semantic and episodic neuropsychological tasks with non-social scene construction converges with current theoretical positions emphasising the interplay between the episodic and semantic memory systems in the rendering of detailed spatial arrays [60]. Notably, we did not find these associations in the social conditions. Rather, socially congruent scene construction was found to correlate exclusively with response inhibition, suggesting a possible role for the suppression of details that do not fit within a given social context. We tentatively propose that the incongruent social condition represents a recombinatorial step too far for patients with dementia, requiring the integration of disparate elements not typically co-located within the same spatial setting or social scenario, in the absence of a suitable event script or schema.

A number of methodological issues warrant consideration in this context. To avoid fatigue in the dementia patients, we limited our study to one trial per condition, thus reducing overall study power. Given our relatively small sample size, we did not run the correlation analyses in the bvFTD and AD groups separately, limiting our capacity to comment on the mechanisms that potentially drive scene construction impairments in these disorders. Future studies in a larger sample of dementia patients, stratified by disease severity, with a greater number of experimental trials will be required to replicate the current findings. Similarly, to ensure a shorter testing time, we opted not to include the Spatial Coherence Index from the original scene construction task. Inclusion of the Spatial Coherence Index would further enable us to determine how manipulations of sociality and congruency influence the spatial cohesion of the constructed scene.

## 5. Conclusions

Our study provides initial clues as to how object–scene (in)congruency impacts the capacity for scene construction in dementia. Despite largely comparable scene construction performance profiles in bvFTD and AD, we suggest that the cognitive and neural mechanisms driving these impairments are likely to differ. For example, constructive deficits in AD might arise due to characteristic episodic and semantic memory disturbances, alongside visuospatial dysfunction [64,65], while deficits in bvFTD might be better explained by socioemotional and executive disturbances that typify this syndrome [32,66]. As we did not include targeted measures of social cognition or emotion processing in this study, future studies will be required to definitively test these proposals. It will also be important to explore how the impairments uncovered in this study relate to social cognitive and behavioural changes, such as apathy, increased mental rigidity and environmental dependency, particularly in bvFTD [67,68]. Finally, we suggest that studies exploring the neural correlates of these disturbances will be important to clarify the respective contribution of key structures implicated in mental construction, most notably the hippocampus and ventromedial prefrontal cortex [30,69]. Addressing these questions will provide important insights regarding the multifaceted processes which enable us to envisage contextually rich scene imagery and how such processes break down in dementia.

## Figures and Tables

**Table 1:** Demographics and clinical characteristics of study participants.

	bvFTD M (SD)	AD M (SD)	Controls M (SD)	Group Effect (F Value)	Post hoc (Direction of Effect)
N	15	11	16	-	-
Sex (M:F)	13:2	6:5	8:8	5.1 ^a^	-
Age (years)	61.4 (9.1)	64.7 (8.4)	64.7 (4.4)	1.0	-
Education (years)	11.7 (2.0)	12.6 (2.6)	14.9 (2.2)	8.0 **	CN > AD, bvFTD
Disease Duration (years)	6.7 (3.5)	4.6 (2.8)	-	0.7 ^b^	-
Disease Severity (CDR-FTLD SoB)	6.3 (3.6)	6.8 (3.3)	-	1.6 ^b^	-
Behavioural Change (CBI-R Total)	38.5 (16.7)	30.9 (17.1)	5.0 (4.0)	21.3 ***	AD, bvFTD > CN
Abnormal Behaviour (CBI-R Sub-scale)	40.6 (22.2)	20.8 (22.7)	3.2 (4.2)	14.3 ***	bvFTD > AD > CN

Notes. ^a^ Chi-square value. ^b^ Independent samples *t*-test. M = mean, SD = standard deviation. Corrected post hoc comparisons are reported. ** = *p* < 0.01, *** = *p* < 0.0001. bvFTD = behavioural-variant frontotemporal dementia; AD = Alzheimer’s disease CN = Controls; CDR-FTLD SoB = Frontotemporal Lobar Degeneration-Modified Clinical Dementia Rating Sum of Boxes score; CBI-R = Cambridge Behavioural Inventory—Revised. Years of Education data unavailable for 1 Control. CBI-R data unavailable for 3 Controls. Disease duration data unavailable for 2 AD and 1 bvFTD.

**Table 2:** Descriptive statistics and group differences on neuropsychological tests.

	bvFTD M (SD)	AD M (SD)	Controls M (SD)	Group Effect (H)	Post Hoc (Direction of Effect)
ACE-III Total (100)	77.7 (6.5)	64.8 (9.8)	95.3 (2.5)	32.8 **	CN > AD, bvFTD
RAVLT 30 min (15)	5.2 (2.8)	1.1 (1.7)	10.8 (1.7)	26.9 **	CN > AD, bvFTD
RCF 3 min (36)	13.2 (6.7)	2.4 (2.0)	16.6 (4.2)	18.5 **	CN > AD; bvFTD > AD
Hayling Overall (7)	4.9 (1.3)	3.6 (0.8)	6.3 (0.7)	18.6 **	CN > AD, bvFTD
Digit Span Forwards	8.9 (2.3)	7.9 (0.7)	11.3 (2.8)	9.8 *	CN > AD
Digit Span Backwards	5.1 (2.0)	3.7 (1.8)	8.3 (3.0)	14.1 *	CN > AD, bvFTD
TMT B-A (seconds)	82.3 (56.7)	142.2 (74.2)	45.7 (14.9)	11.4 *	CN < AD
SydBat Naming (30)	24.1 (2.3)	19.6 (4.9)	27.1 (2.3)	16.0 **	CN > AD
SydBat Semantic (30)	27.0 (2.0)	24.3 (2.3)	28.6 (1.0)	18.8 **	CN > AD
ACE-Language (26)	23.3 (2.5)	21.7 (2.8)	25.5 (0.8)	18.1 **	CN > AD, bvFTD
ACE-Fluency (14)	8.6 (2.3)	8.0 (2.6)	11.9 (1.6)	19.0 **	CN > AD, bvFTD

Notes. Maximum test scores shown in brackets where appropriate. M = mean. SD = standard deviation. Corrected post hoc comparisons shown. * = *p* < 0.01, ** = *p* < 0.0001. bvFTD = behavioural-variant frontotemporal dementia; AD = Alzheimer’s disease, CN = Controls; ACE-III = Addenbrooke’s Cognitive Examination—Third Edition; RAVLT = Rey Auditory Verbal Learning Test; RCF = Rey Complex Figure; Hayling overall scaled score; TMT B-A = Trail Making Test—Time B minus Time A; SydBat = Sydney Language Battery. Data unavailable for the following tests: RAVLT: 2 CN, 4 AD; RCF: 2 CN, 3 AD; Hayling: 2 CN, 4 AD, 1 bvFTD; TMT B-A: 2 CN, 6 AD, 2 bvFTD.

**Table 3:** Subjective ratings for each condition in participant groups.

			bvFTD M (SD)	AD M (SD)	Control M (SD)
Difficulty	Congruent	Social	2.1 (0.9)	2.5 (0.9)	2.2 (1.1)
		Non-Social	2.9 (1.1)	2.6 (1.1)	2.3 (0.9)
	Incongruent	Social	3.0 (1.1)	2.7 (1.0)	2.4 (1.0)
		Non-Social	2.7 (1.2)	3.4 (1.1)	2.6 (1.3)
Vividness	Congruent	Social	3.2 (0.9)	3.6 (0.7)	3.7 (0.8)
		Non-Social	3.1 (1.0)	3.5 (0.7)	3.6 (1.0)
	Incongruent	Social	3.1 (1.1)	3.1 (0.9)	3.6 (1.0)
		Non-Social	3.3 (1.0)	3.6 (0.9)	3.3 (1.1)
Level of Detail	Congruent	Social Non-Social	3.3 (0.9) 3.1 (1.0)	2.9 (0.8) 2.7 (0.8)	3.4 (0.8) 3.4 (0.8)
	Incongruent	Social	3.0 (1.1)	2.7 (0.6)	3.3 (0.8)
		Non-Social	3.4 (1.0)	2.9 (0.9)	3.0 (0.9)
Sense of Presence	Congruent	Social Non-Social	3.5 (0.9) 3.2 (1.1)	4.0 (0.6) 3.5 (0.8)	3.9 (1.0) 3.6 (1.1)
	Incongruent	Social	3.1 (1.1)	3.4 (0.9)	3.5 (1.1)
		Non-Social	3.3 (1.2)	3.9 (0.9)	3.4 (1.1)
Realism	Congruent	Social	3.1 (1.0)	3.1 (0.6)	3.3 (1.1)
		Non-Social	2.9 (0.9)	3.3 (0.9)	3.4 (1.0)
	Incongruent	Social	3.1 (1.1)	3.4 (0.9)	3.5 (1.1)
		Non-Social	3.3 (1.2)	3.9 (0.9)	3.4 (1.1)
Similar to Memory	Congruent	Social Non-Social	2.8 (1.1) 3.1 (1.3)	2.6 (1.5) 2.4 (0.8)	3.3 (1.0) 3.1 (1.1)
	Incongruent	Social	4.0 (1.0)	4.0 (1.1)	3.9 (1.4)
		Non-Social	4.3 (0.8)	3.8 (1.1)	4.1 (1.4)

Notes. M = mean. SD = standard deviation. For all ratings higher scores = stronger perceived experience, i.e., greater difficulty; more vividness; richer detail; more realistic; except similarity to memory where lower scores = more similar to a previous memory. bvFTD = behavioural variant of frontotemporal dementia. AD = Alzheimer’s disease.

**Table 4:** Pearson correlation coefficients exploring associations between scene construction performance and cognitive variables in AD and bvFTD groups combined (*n* = 23).

	Total Content Scores			
	Congruent		Incongruent	
	Social	Non-Social	Social	Non-Social
SydBat Semantic Association	0.237	0.391 *	0.005	0.483 *
SydBat Semantic Naming	−0.181	0.427 *	0.014	0.331
RCF 3 min recall	0.347	0.411 *	0.214	0.528 **
Hayling Overall Scaled	0.393 *	0.275	0.129	0.333
RAVLT 30 min recall	−0.143	0.090	0.261	0.177

Notes. Uncorrected one-tailed Pearson correlation coefficients. * = *p* < 0.05 ** = *p* ≤ 0.01. Patient numbers within each test: SydBat = Sydney Language Battery, AD = 11, bvFTD = 9; RCF= Rey Complex Figure, AD = 8, bvFTD = 15; Hayling, AD = 6, bvFTD = 14; RAVLT = Rey Auditory Verbal Learning Test, AD = 7, bvFTD = 15.

## Data Availability

The ethical requirement to ensure patient confidentiality precludes public archiving of our data. Researchers who would like to access the raw data should contact the corresponding author, who will liaise with the ethics committee that approved the study. Accordingly, as much data as are required to reproduce the results will be released to the individual researcher. No parts of the study procedures or analyses were registered prior to the research being undertaken.

## Supplementary Materials

The following are available online at https://www.mdpi.com/article/10.3390/brainsci12010020/s1, Supplementary Material: Table S1: Pilot ratings of item congruence for scene-object cues on the modified scene construction task; Supplementary Material 2: Sample scene description provided to participants by the experimenter; Supplementary Material 3: Prompting instructions provided to participants during scene construction.
