# Supplementary material for: Putting the Pieces Together: Mental Construction of Semantically Congruent and Incongruent Scenes in Dementia
Source: Brain Sci. 2021 Dec 24;12(1):20. doi: 10.3390/brainsci12010020 (PMC8773466; doi:10.3390/brainsci12010020)
Supplement: Supplementary file 1 [file brainsci-12-00020-s001.zip › brainsci-1512515-supplementary.pdf]

Putting the pieces together: Mental construction of semantically congruent and incongruent scenes in dementia

Nikki-Anne Wilson[1,2,3,4]; Rebekah M. Ahmed[1,5]; Olivier Piguet[1,2]; Muireann Irish[1,2*]

[1] The University of Sydney, Brain and Mind Centre, Sydney, NSW 2050, Australia
[2] The University of Sydney, School of Psychology, Sydney, NSW 2006, Australia
[3] The University of New South Wales, School of Psychology, Sydney, NSW 2052, Australia
[4] Neuroscience Research Australia, Sydney, NSW 2031, Australia
[5] Memory and Cognition Clinic, Institute of Clinical Neurosciences, Royal Prince Alfred Hospital, Sydney, NSW 2050, Australia

* Professor Muireann Irish, Brain and Mind Centre, The University of Sydney, NSW 2050. Email: muireann.irish@sydney.edu.au and Dr Nikki-Anne Wilson, Neuroscience Research Australia, Randwick, NSW, 2031.Email: n.wilson@neura.edu.au

Table S1. Pilot ratings of item congruence for scene-object cues on the modified scene construction task.

ID	Beach & Ice-Skates	Clown & Funeral	Classroom & Books	Hospital & Doctor
1	1	1	7	7
2	1	1	7	7
3	1	1	7	7
4	1	1	7	7
5	1	1	7	7
6	1	1	7	7
7	1	1	7	7
8	1	1	7	7
9	1	1	7	7
10	1	1	7	7

Note. A group of N=10 healthy, young adults rated each of the items as to "How well the two elements went together" where 1 = did not go together at all; 7 = went together very well.

Supplementary Material 2. Sample scene description provided to participants by the experimenter.

I'm going to give you an example with two things that don't feel like they belong together. "You're in a boardroom. There is a hairdryer there". So I could say:

> *"I'm standing in an impressive office boardroom in a city skyscraper. Out the window I can see the whole city! I'm excited as I'm here to launch a national advertising campaign for my new hairdryer. The room smells of coffee – I'm already on to my third cup and now I'm not sure if its nerves or caffeine I can feel! They've told me that they've secured a big celebrity for the launch – I can hear voices approaching from outside now – I can't wait to see who it is!"*

See how I included the hairdryer into the boardroom scene, even though those aren't two things which would normally belong together? I'm going to give you 2 minutes to describe each scene and it's really important that you try to keep talking for the whole 2 minutes.

Supplementary Material 3. Prompting instructions provided to participants during scene construction.

If participants are stuck and fail to say anything for more than 15-30 seconds OR if they only include ONE of the cue elements, the following general prompts are to be provided to encourage further details:

Prompt 1: 'Remembering to include both the X and the Y in the scenario that you're describing, are there any other details you can tell me?

If still nothing after another 15-30 seconds, offer a second general prompt:

Prompt 2: 'As you picture the scenario with the X and the Y, is there anything else you can describe to me?'

If participant is still unable to provide any details, the trial should be abandoned, noting the time of ending.
